# Does Incorporation of the Dorsal Cutaneous Branch of the Proper Digital Nerve in Anterograde Neurovascular Island Flap Improve Outcomes? Results of A Retrospective Case Series

**DOI:** 10.1055/a-2690-1599

**Published:** 2025-11-20

**Authors:** Pervaiz M. Hashmi, Abeer Musaddique, Muhammad Ali, Muhammad A.A. Khan, Muhammad Z.N. Khan, Marij Zahid

**Affiliations:** 1 Section of Orthopedics, Department of Surgery, Aga Khan University Hospital, Karachi, Pakistan; 2 School of Nursing and Midwifery, Aga Khan University Hospital, Karachi, Pakistan; 3 Department of Public Health, University of Bradford, Bradford, United Kingdom; 4 Department of Orthopedic Surgery, Hamdard College of Medicine and Dentistry, Karachi, Pakistan; 5 Department of Orthopedic Surgery, Aga Khan University Hospital, Karachi, Pakistan

**Keywords:** neurovascular island flap, fingertip injuries, anterograde flap, pulp loss, fingertip injuries

## Abstract

**Background:**

Fingertip injuries present a challenge for surgeons. Factors such as appearance, sensation, and grip strength influence the choice of procedure. The neurovascular island (NVI) flap is commonly used due to its reliability and favorable results. However, it can lead to reduced sensation and a limited range of motion, which are significant concerns.

**Methods:**

This study analyzed 56 adult patients with 66 finger injuries treated with a modified NVI flap by a single surgeon. The flap is based on branches of the proper digital artery to the volar and dorsal skin, including two nerves in the resected flap. The FIOS score was used to assess long-term flap outcomes at follow-up. Patients with thumb injuries, incomplete records, or loss of follow-up were excluded.

**Results:**

The study found that NVI flaps had a survival rate of 98%. Post-surgery, the patients' outcomes were assessed using the FIOS score, with 61 cases rated as “excellent,” 4 as “good,” and 1 as “fair.” During long-term follow-up, none of the patients experienced cold intolerance or sensory abnormalities, which is attributed to the inclusion of the dorsal cutaneous nerve in the flap.

**Conclusion:**

In conclusion, the NVI flap procedure proved to be a reliable method for reconstructing fingertip pulp loss, offering long-term satisfactory outcomes. It provided good coverage with preserved sensation, dexterity, and preservation of finger length, meeting the patients' cosmetic expectations.

## Introduction

Fingertip injuries lead to considerable morbidity, causing pain, loss of work and leisure time, financial strain, and changes to body appearance. 1 In the United States alone, the annual incidence of digital injury rate is twice that of the shoulder and wrist injury rate, which is 444, 200, and 181 per 100,000 patients, respectively. 2 Clinical presentation of fingertip injuries ranges from minor nail bed injury and pulp loss to traumatic amputation of fingers. These clinical presentations need immediate treatment to avoid any temporary or permanent disability. 3 4 Depending upon the injury severity, as mentioned by Allen 5 and Lim's classification, 6 treatment ranges from simple laceration repair to soft tissue reconstruction.

Soft tissue reconstruction of injured fingertips is challenging, requiring careful attention to functionality, cosmesis, and sensation. 7 8 Various methods yield good results, including local (V–Y advancement flap by Atasoy, homodigital anterograde neurovascular island [NVI] flap), regional (cross-digit, retrograde NVI flap), and distant and free flaps. The NVI flap, first defined by Bunnel 9 and later modified by Segmuller, 10 involves mobilizing a neurovascular bundle to enhance flap excursion and using an oblique triangular flap for oblique fingertip amputations. Literature on the best reconstruction technique is conflicted, often depending on the surgeon's preference, expertise, and patient's cosmetic desires. However, the anterograde homodigital NVI flap has a lower complication rate (3.8%) compared with retrograde NVI (8.0%) and cross-finger flaps (6.3%), making it a preferred method for many surgeons. Flap resection techniques vary, especially in flap and pedicle length. 10

Our study aimed to assess the long-term functional outcomes and immediate complications of a modified NVI flap technique. This technique incorporates the dorsal digital branch of the proper digital nerve, enhancing flap recovery and sensitivity by including equal amounts of volar and dorsal skin.

## Methods

We performed a retrospective case series of 56 patients with 66 finger flaps from July 1996 to February 2020. We included all adult patients who underwent a distally based modified NVI flap for fingers by a single surgeon in the above-specified time frame using the same standardized technique. Patients having thumb injuries, incomplete medical records, and loss of follow-up were excluded from the study. The study has been approved by the hospital's Ethical Review Committee (2020-5329-14408). Data from these patients were collected from the hospital information management system and electronic medical records, which included demographic parameters, mechanism of injury, defect site and size, size of NVI flap, hospital stay, postoperative course, immediate complications, and functional outcome of the flap quantified by FIOS scoring. All patients provided informed consent for the utilization of their surgical and hand images in this research.

Immediate complications were defined as all complications incurred after flap surgery during the hospital stay, including flap failure, tip necrosis, partial flap necrosis, and venous congestion. Long-term flap outcomes were assessed using the Fingertip Injuries Outcome Score (FIOS). The FIOS was administered according to patients' follow-up and consisted mainly of 10 Likert-type questions. 11 The questions included objective measures like nail and finger length, bone health, sensation, range of motion, and grip strength, as well as subjective measures such as pain, cosmesis, and return to work. A total score less than 12 was considered excellent, scores ranging from 13 to 18 were considered good, scores ranging from 19 to 24 were considered fair, and any score greater than 24 was marked as poor.

Categorical variables like gender, mechanism of injury, defect site, size, etc., were recorded as frequencies and percentages. Discrete and continuous variables like age and defect size were recorded as mean ± standard deviation (SD), or median with interquartile ranges where appropriate.

### Basis of Flap

**Fig. 1:**
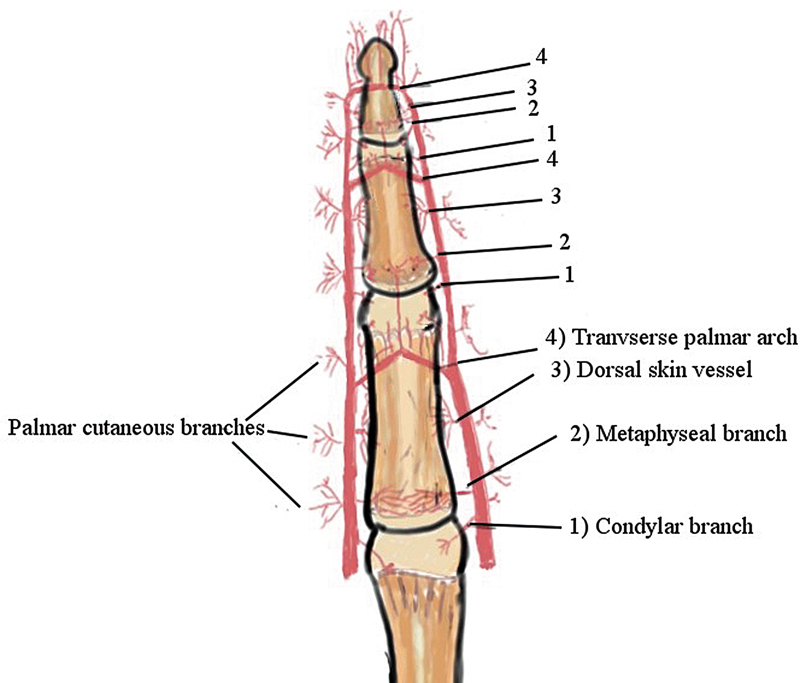
Shows the diagrammatic representation of the branches of the digital artery as observed in our cadaveric dissections.

The modified NVI flap consists of an elliptical or triangular paddle of skin with a pedicle containing the proper digital artery, the nerve, and its dorsal cutaneous branch. The proper digital artery along the course gives multiple branches at each phalanx at the palmar and dorsal levels. Figure 1 shows a diagrammatic representation of the dorsal and palmar branches, as shown in our dissections. A similar network of arteries was also reported in the novel study by Strauch et al. 12

Each digit is innervated by two proper digital nerves on the radial and ulnar sides of the finger. Each proper digital nerve gives a cutaneous branch at the level of the proximal phalanx that crosses the proximal interphalangeal (PIP) joint to give sensation to the dorsal skin of the middle and distal phalanx. When the flap is dissected, care is taken to include the arterial branches from both the palmar and the dorsal sides of the digit, ensuring maximum viability of the flap.

### Surgical Technique

**Fig. 2:**
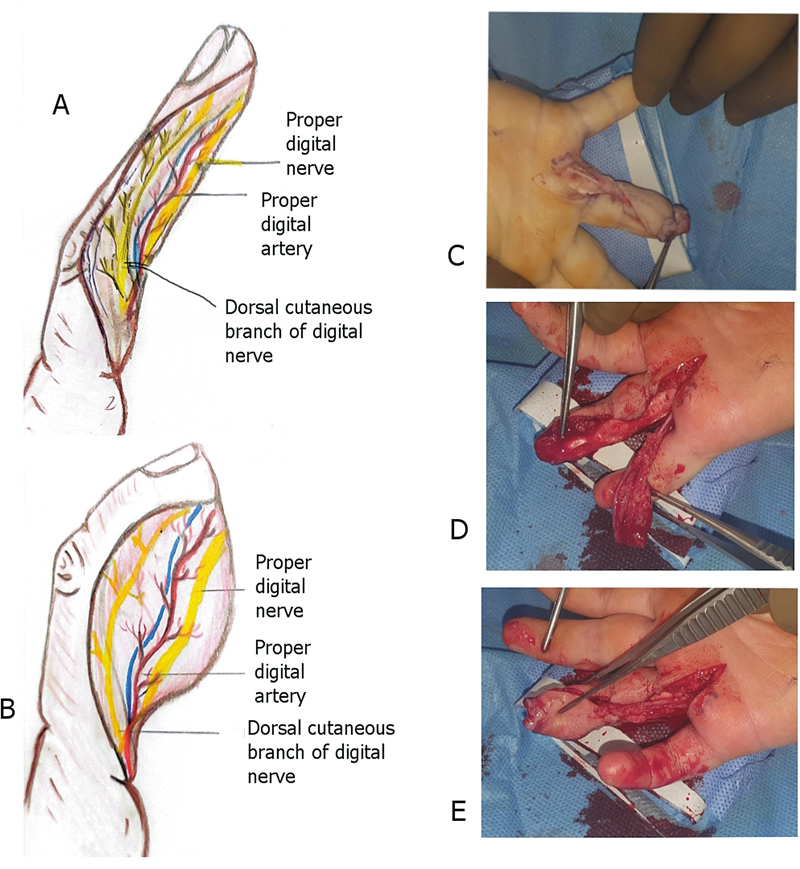
**(A)** Shows the marking of the flap incorporating the dorsal half of the skin over the distal interphalangeal joint and PIPJ, the volar half of the skin. The flap incorporates the dorsal cutaneous branch of the proper digital nerve (sensory supply of dorsal skin) and the proper neurovascular bundle. **(B)** Shows a closer view of the flap incorporating the neurovascular bundle, especially the dorsal cutaneous nerve. **(C)** Shows the dissected flap after tourniquet placement. **(D, E)** Shows the flap after tourniquet removal.

After exsanguination with a crepe bandage or simple elevation of the hand/upper extremity and inflation of the tourniquet, the flap is outlined as a triangular piece of flap with its volar margins in the midline of the finger and dorsal margin almost in the mid-dorsum of the finger. The proximal limit of the flap extends up to or proximal to the PIP joint to incorporate the dorsal cutaneous branch of the proper digital nerve. The width of the flap includes almost half of the volar and dorsal skin. The length of the flap is variable, and it usually extends beyond the PIP joint, where both volar and dorsal incisions meet across the proximal phalanx. After the volar incision is made, the neurovascular bundle with the flap is lifted over the flexor sheath, protecting it and its contents. After the dorsal incision is made, the flap is raised over the epitenon of the extensor tendon. During the dissection under magnification, monopolar cautery is used to coagulate the small vessels, including the metaphyseal, diaphyseal, and vincular branches going to the flexor sheath. Once the flap is raised, the main digital neurovascular bundle is dissected through a mid-lateral incision in the finger that can be extended to the palm to get the extra length of the neurovascular bundle. The incision must be made in a manner that incorporates the dorsal cutaneous branch of the proper digital nerve. The distal reach of the flap can be improved with proximal dissection of the neurovascular bundle in the palm and flexion of fingers at the metaphyseal and PIP joints. It is to be noted that there is no ligation of the proximal end of the neurovascular bundle. The flap is merely advanced to cover the more distal defect. The size of the flap is variable and can be 1.5 cm wide to 3.5 cm long, depending upon the defect size. The digital pedicle is dissected with the surrounding fatty tissues because the venae comitantes are small, hard to define, and run in this fat. 13 It is essential to safeguard the venous drainage of the flap. Advancement is obtained by gentle traction on the pedicle. It is possible because of the freeing of soft tissues around the pedicle, the natural slight elasticity of the pedicle, and the medial migration of the pedicle when using a radial island flap on the index finger. The radial digital bundle forms an angle at the level of the metacarpal head, and by converting this to a straight line, an advancement of 1.7 cm ^2^ can be obtained. In my experience, I have used up to 2.5 cm of defect size ( Fig. 2A – D ).

### Cases

#### Case 1

**Fig. 3:**
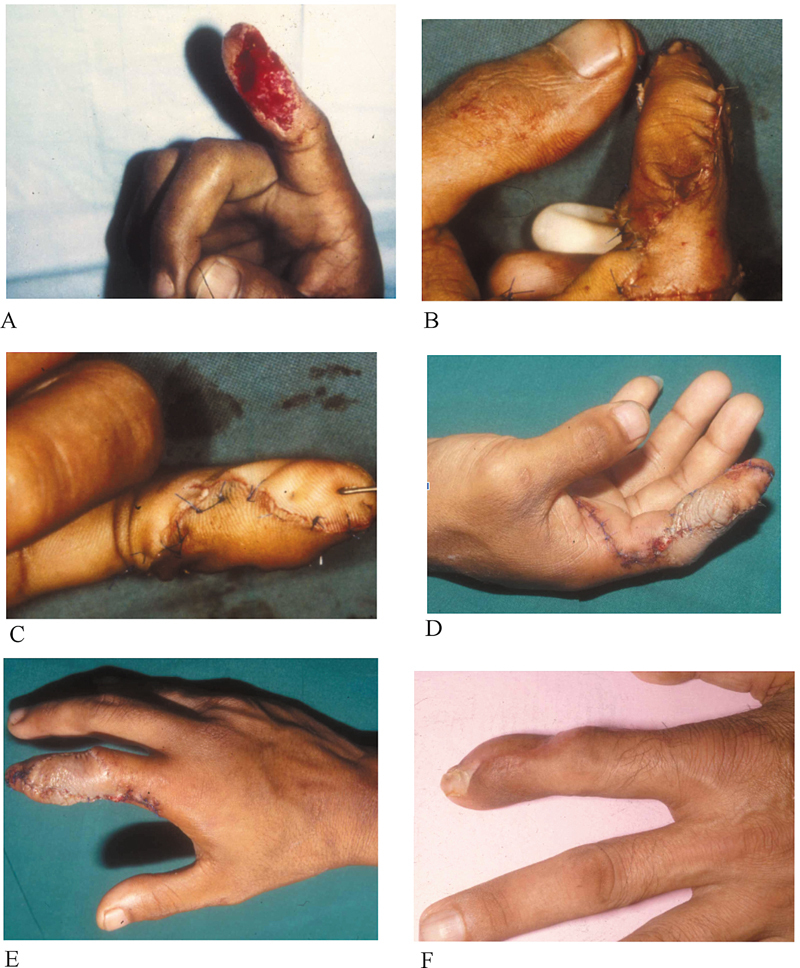
**(A)** Shows the extent of injury, and **(B, C)** shows intraoperative photographs after the defect was repaired. **(D)** Shows a defect 1 week after the repair. **(E, F)** Photographs 12 weeks after the repair.

A 34-year-old man sustained a glass cut injury to the suitable index finger pulp on the palmar side. The defect measured 3 × 1.5 cm ( Fig. 3A ). He underwent a homodigital NVI flap from his radial side with excellent coverage and cosmesis 20 weeks post-surgery. No postoperative complications were observed ( Fig. 3 ).

#### Case 2

**Fig. 4:**
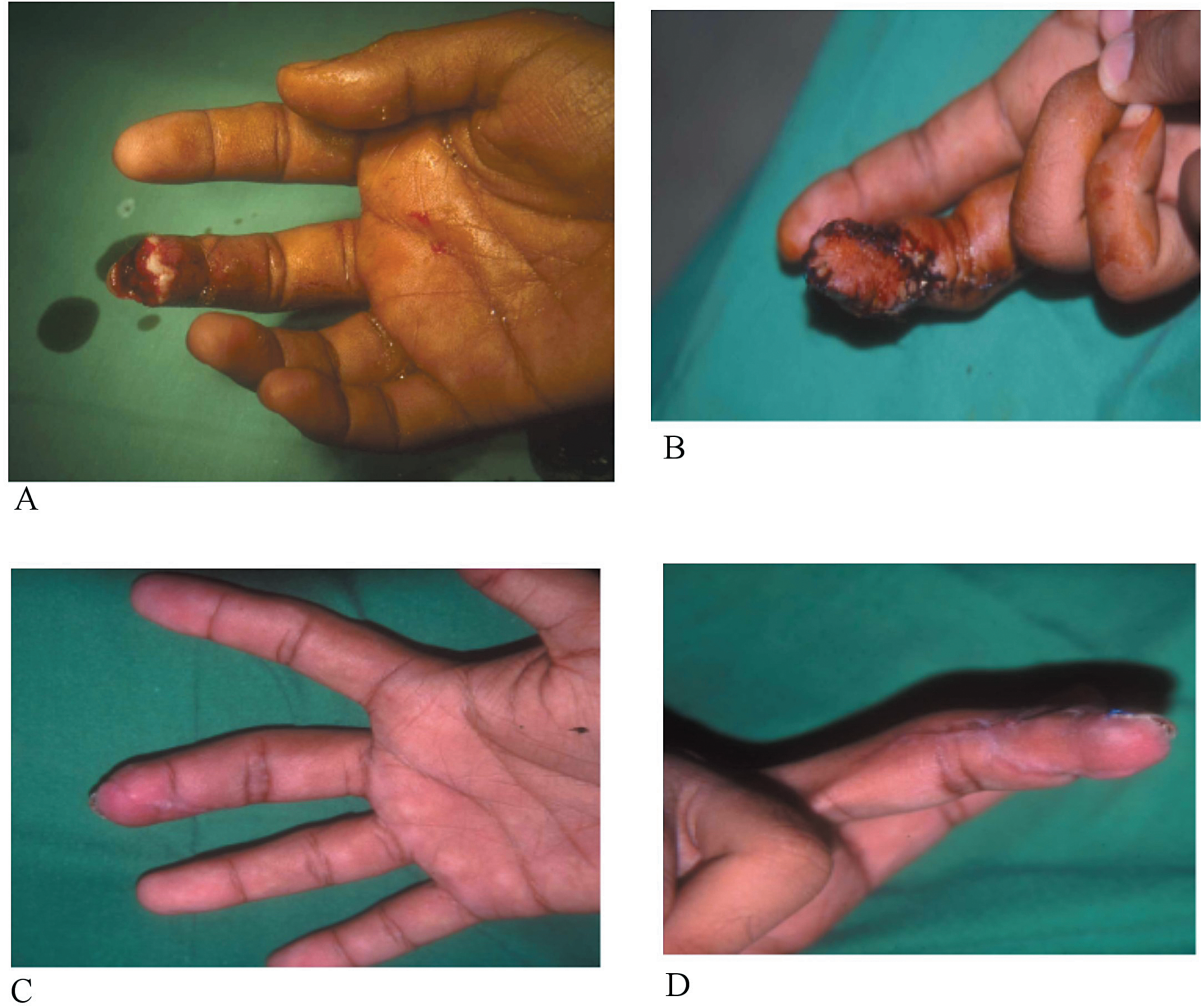
**(A)** Shows the extent of injury, and **(B)** shows an intraoperative photograph after surgery. **(C, D)** Photographs 4 weeks after the repair.

A 36-year-old man sustained a table saw injury to the right middle finger pulp with an intact nail bed. His defect size was 2 × 1.5 cm ( Fig. 4A ). He also underwent NVI flap placement. A flap was lifted from the ulnar side of the same digit ( Fig. 4B ). Figure 4C , D shows the same finger 16 weeks post-repair. No complications were noted, and the patient was satisfied with the outcome.

## Results

From 1996 to 2020, 56 patients underwent homodigital modified NVI flap reconstruction (66 flaps). There were 47 males (84%) and 9 females (16%) with a mean age of 38.75 (SD 14.79). Both hands were equally injured (50%). Among injuries of the digits, the index (38%) and middle fingers (30.3%) were most often injured, and most cases (84%) involved a single digit. Of all the fingertip injuries that were repaired via homodigital modified NVI flap, traumatic injuries made up the bulk of fingertip injuries (78.6%). This included machine cut injuries 32 (57.1%), door entrapments 8 (14.3%), and road traffic accident 4 (7.1%; Table 1 ).

**Table 1:** Depicts the demographic characteristics of the included population

S.No.	Variables	Number (percentage)	
1	Gender	Male Female	47 (84) 9 (16)
2	Age (years)	Mean (SD)	38.75 ± 14.79
3	Mechanism of injury	Traumatic	44 (78.6)
		Burn injury	4 (7.1)
		Infection	6 (10.7)
		Contracture	1 (1.8)
		Tumor	1 (1.8)
4	Defect side	Right Left	28 (50) 28 (50)
5	Digits involved	Index finger	25 (38)
		Middle finger	20 (30.3)
		Ring finger	15 (22.7)
		Little finger	7 (9)
6	Defect size	Mean in cm (L + B)/2	1.68 ± 0.28
7	Immediate complications	Flap failure	1 (1.5)
		Partial necrosis	1 (1.5)
		Tip + partial necrosis	2 (both fingers; 3)
		Venous congestion	1 (1.5)
		No complications	61 (92)
8	Flap survival	65 (98.5)	

Abbreviation: SD, standard deviation.

Out of 66 flaps, 61 (92%) showed complete healing with no complications. Flap complications were recorded in only five (8%) cases. One patient developed complete necrosis and subsequently underwent groin flap placement; one patient developed tip necrosis, and two fingers developed partial epidermal necrosis, which required subsequent daily dressing to re-epithelize. One patient had venous congestion that recovered without skin necrosis.

The mean defect size of the finger was 2.62 + 0.43 cm ^2^ . All these patients were followed up for 2 to 12 years (mean: 6.3 [SD 2.2]) in the clinic until complete healing. Overall, the flap survival rate was 98% with complete primary healing.

**Fig. 5:**
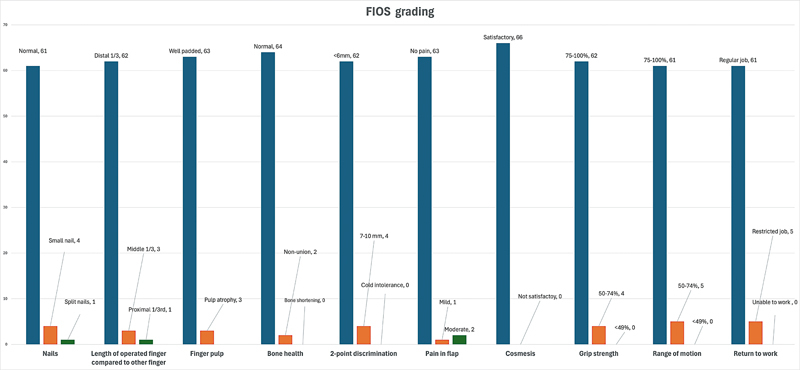
Shows the individual components of FIOS and their respective frequencies. FIOS, Fingertip Injuries Outcome Score.

We used the FIOS to assess the cosmesis and functional outcome of the flaps. Out of 66 flaps, 61 (92.4%) flaps were graded as “excellent,” 4 (6.1%) were graded as “good,” and 1 (1.5%) flap was graded as “fair.” A total of 10 flap characteristics were assessed in the FIOS score. Three patients developed parrot beak nail deformity, and three patients had finger pulp atrophy. Most phalanges healed perfectly, except for two that went into non-union. Patient satisfaction with finger flap cosmesis varied, with all patients except one being happy with their surgery. In terms of sensation, two-point discrimination was normal in 62 cases, with 4 patients experiencing hyperesthesia. The majority of patients remained pain-free, but a few reported mild to moderate pain. Additionally, some patients experienced a slightly decreased range of motion and finger grip strength. Table 2 summarizes the results of FIOS classification. Figure 5 depicts the individual components of the scoring system.

**Fig. 6:**
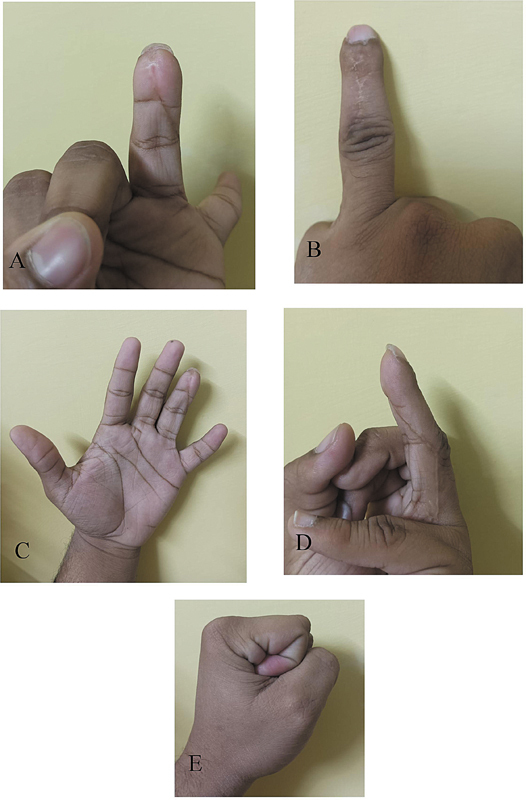
**(A–E)** Shows the appearance of the healed flap in different positions 1 year after surgery.

**Table 2:** Summarizes the results of Fingertip Injuries Outcome Score classification

Grading	Excellent	Good	Fair
Frequency (%)	61 (92.4)	4 (6.1)	1 (1.5)

## Discussion

Despite many options, choosing a suitable treatment for an individual patient with fingertip injuries remains challenging. 14 The modified homodigital island flap meets many of the criteria for an ideal reconstruction, yet only a few studies have been published with a longer follow-up. Knowing the long-term outcome would help surgeons and patients make an informed decision when considering the right option for the individual patient.

Our study reported that 78.6% of the fingertip injuries were due to trauma. This is consistent with a similar study published in India, which reported a rate of 40.1%. 15 Pakistan and India likewise have a large population from low socioeconomic backgrounds and, therefore, have a more significant propensity to encounter traumatic mechanical injuries.

The findings of the current study indicate that these patients generally experienced favorable functional outcomes from the flap surgery, maintaining satisfactory digit functionality over the 10 years following the procedure. Foucher et al reported two cases (3%) with total flap loss in their cohort of 64 NVI flaps. 16 We reported one case (2%) with flap failure. This patient presented to us after 2 weeks of sustaining an injury and had multiple comorbidities.

Venkataswami and Subramanian, in their original description of the “oblique, triangular flap,” reported results similar to ours in their cohort of 78 patients. 17 No flaps were lost completely in their series, but four (5%) patients had marginal necrosis of the flap. In our study, flap necrosis was observed in only three (5%) cases.

FIOS scoring was used to assess the long-term outcomes of the modified flaps in our study. Sixty-one (92.4%) patients had excellent grading, indicating that the anterograde homodigital NVI flap offers good long-term outcomes. The superiority of homodigital anterograde NVI lies in its ability to be fully sensate flaps with minimal failure rates ( Fig. 6 ).

The major long-term complication found in our study was parrot beak deformity in three patients (4.5%). Parrot beak deformity is a reasonably common observed complication. While meticulous surgery can minimize the risk, variations in wound healing can make parrot beaking unavoidable in a set of patients. In another study by Kayalar et al, 7 parrot beak deformity was reported to be 7%, which is comparable to our study. Braga-Silva et al compared anterograde NVI flaps to retrograde NVI flaps in a randomized prospective study in which anterograde NVI showed better results in terms of discriminatory sensation ( *p* < 0.001) and overall flap viability. 18 However, a systematic review published on the efficacy of anterograde NVI flap showed the major complication to be cold intolerance at 18%. 19 We had no patients with cold intolerance. This can be one of the limitations of our study, as in our part of the country, there is a short duration of winters of hardly 3 months during which the temperature does not drop below 20 °C and we could not experience any case of cold insensitivity. However, four of our patients did experience hyperesthesia and this can be attributed to the inclusion of two nerves in the flap: The palmar digital nerve in the volar side of the flap and the dorsal cutaneous branch from the dorsal part of the flap.

In our study, skin contracture was seen in only two (4%) patients, which is lower than in other similar studies. Kayalar et al 7 reported nine (11%) cases, while Lok et al 20 reported five (38%) cases of flexion contracture. This difference could be attributed to adequate advancement of the NVI flap in our technique, which prevents flexion contracture in the digits.

Our studies had various limitations. Due to the retrospective nature of our study, the time to follow up for FIOS scoring was variable. Furthermore, the data reported on the cause of fingertip injuries only represented the patients who underwent flap surgery.

### Conclusion

Our study demonstrates that the modified NVI flap technique offers excellent long-term functional outcomes and minimal immediate complications for fingertip injury reconstruction owing to its sensate properties, improved grip strength, and greater range of motion. With a 98% flap survival rate and a 92.4% excellent FIOS grading, this technique proves to be highly effective. The inclusion of both volar and dorsal skin, along with the dorsal digital branch of the proper digital nerve, enhances flap recovery and sensitivity. Most patients maintained satisfactory digit functionality, with minimal long-term complications, such as parrot beak deformity and skin contracture.
